# Supplementary material for: Three-Dimensional Comparison of the Maxillary Surfaces through ICP-Type Algorithm: Accuracy Evaluation of CAD/CAM Technologies in Orthognathic Surgery
Source: Int J Environ Res Public Health. 2022 Sep 19;19(18):11834. doi: 10.3390/ijerph191811834 (PMC9517090; doi:10.3390/ijerph191811834)
Supplement: Supplementary file 1 [file ijerph-19-11834-s001.zip › ijerph-1867871-supplementary.pdf]

International Journal of
Environmental Research and Public Health

MDPI

Supplementary Materials

Supplementary Table S1. Patient information included in the study.

Patient	Gender	Age (M: 29,7)	MMD	Surgical Treatment	CAD/CAM System	Type of Osteotomy
1	F	33	III, CH	1, 2, 3	P	MO
2	F	32	II, OB	1, 2	P	SO
3	M	53	III, CH	1, 2, 3	P	MO
4	M	27	II, OB	1,2, 4	P	MO
5	M	26	II	1, 2	P	SO
6	F	25	II, OB	1, 2	S	SO
7	M	23	III	1, 2, 4	S	MO
8	F	19	III	1, 2	S	MO
9	F	32	III	1, 2	S	SO
10	M	27	III, CH	1, 2, 3, 4	S	MO

Legend: MMD (Maxillo-Mandibular Disease): II = Class II malocclusion, III = Class III malocclusion, OB = Open-Bite, CH = condylar hyperplasia; Surgical treatment: 1 = Le Fort I osteotomy, 2 = bilateral sagittal split osteotomy, 3 = condilectomy, 4 = genioplasty; CAD/CAM system: P = PSIs (Splintless– cutting guides and custom-made plates); S = occlusal splints; Type of Maxillary Osteotomy: MO = monobloc (Le Fort I), SO = segmental osteotomy.

Supplementary Table S2. Summary of the 3D comparison (RMSE) in the three groups analyzed.

	Splintless (RMSE)	SD	Splint (RMSE)	SD	*p*-Value
**Total**	1.2167	0.456	1.633	0.303	0.09
**Monobloc Osteotomy**	0.9194	0.062	1.7839	0.311	**0.02**
**Segmental Osteot-omy**	1.6627	0.405	1.4068	0.069	0.23
